# Supplementary material for: Aerobic and Anaerobic Bacterial and Fungal Degradation of Pyrene: Mechanism Pathway Including Biochemical Reaction and Catabolic Genes
Source: Int J Mol Sci. 2021 Jul 30;22(15):8202. doi: 10.3390/ijms22158202 (PMC8347714; doi:10.3390/ijms22158202)
Supplement: Supplementary file 1 [file ijms-22-08202-s001.zip › ijms-1292579-supplementary.pdf]

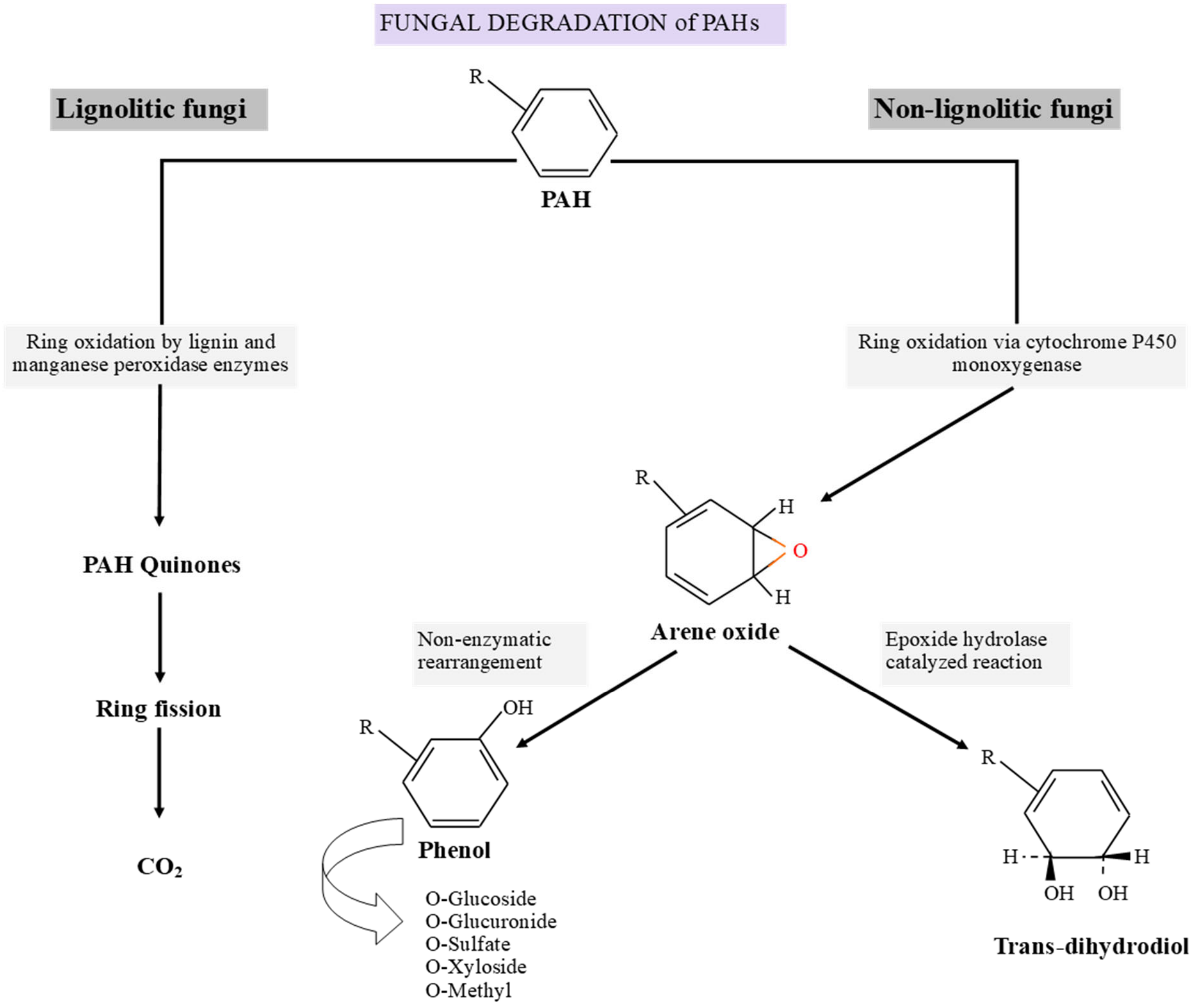


Figure S1: The three pathways used by fungi to metabolize PAHs

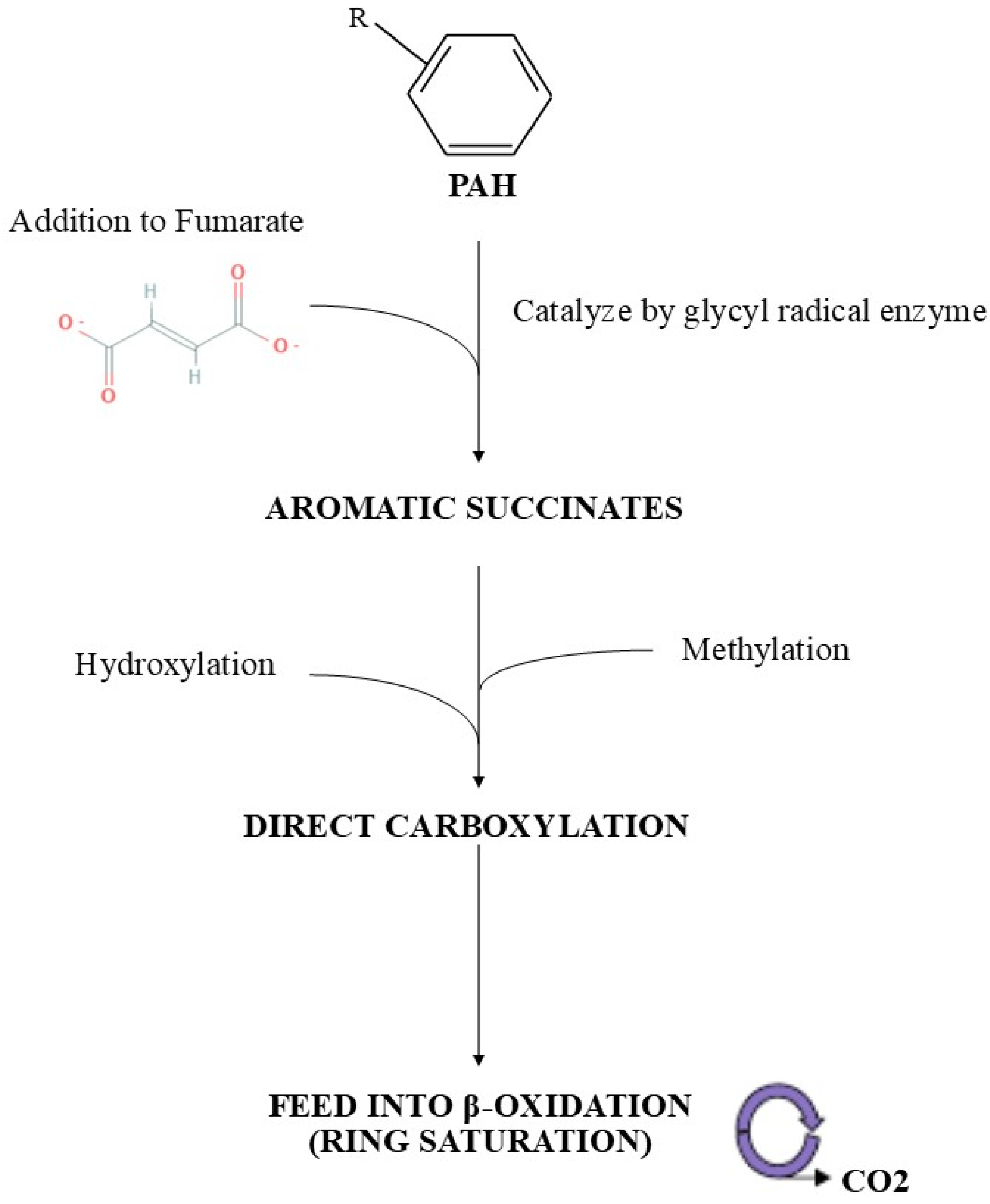


Figure S2: simplified representation of PAHs anaerobic degradation by fungi
